# The Duchenne muscular dystrophy gene and cancer

**DOI:** 10.1007/s13402-020-00572-y

**Published:** 2020-11-14

**Authors:** Leanne Jones, Michael Naidoo, Lee R. Machado, Karen Anthony

**Affiliations:** 1 Centre for Physical Activity and Life Sciences, University of Northampton, University Drive, Northampton, NN1 5PH UK; 2 Department of Genetics and Genome Science, University of Leicester, LE1 7RH Leicester, UK

**Keywords:** Duchenne muscular dystrophy, DMD, Dp71, Dystrophin, Cancer

## Abstract

**Background:**

Mutation of the Duchenne muscular dystrophy (*DMD)* gene causes Duchenne and Becker muscular dystrophy, degenerative neuromuscular disorders that primarily affect voluntary muscles. However, increasing evidence implicates *DMD* in the development of all major cancer types. *DMD* is a large gene with 79 exons that codes for the essential muscle protein dystrophin. Alternative promotor usage drives the production of several additional dystrophin protein products with roles that extend beyond skeletal muscle. The importance and function(s) of these gene products outside of muscle are not well understood.

**Conclusions:**

We highlight a clear role for *DMD* in the pathogenesis of several cancers, including sarcomas, leukaemia’s, lymphomas, nervous system tumours, melanomas and various carcinomas. We note that the normal balance of *DMD* gene products is often disrupted in cancer. The short dystrophin protein Dp71 is, for example, typically maintained in cancer whilst the full-length Dp427 gene product, a likely tumour suppressor, is frequently inactivated in cancer due to a recurrent loss of 5’ exons. Therefore, the ratio of short and long gene products may be important in tumorigenesis. In this review, we summarise the tumours in which *DMD* is implicated and provide a hypothesis for possible mechanisms of tumorigenesis, although the question of cause or effect may remain. We hope to stimulate further study into the potential role of *DMD* gene products in cancer and the development of novel therapeutics that target *DMD*.

## Introduction

The Duchenne muscular dystrophy gene (*DMD*) is best known for its role in the disease of the same name [1]. *DMD* encodes dystrophin protein (Dp) products which are named based on their length in kDa. The major, and full-length, product is the 427 kDa dystrophin protein (Dp427) predominantly expressed in skeletal muscle [1]. Dp427 is essential for maintaining muscle integrity through connecting the actin cytoskeleton to the extracellular matrix. It achieves this through association with various proteins to form a transmembrane scaffolding complex called the dystrophin-associated protein complex (DAPC). The loss of dystrophin, through out-of-frame *DMD* gene mutation, in Duchenne patients is responsible for severe muscle degeneration and premature death [1].

**Fig. 1:**
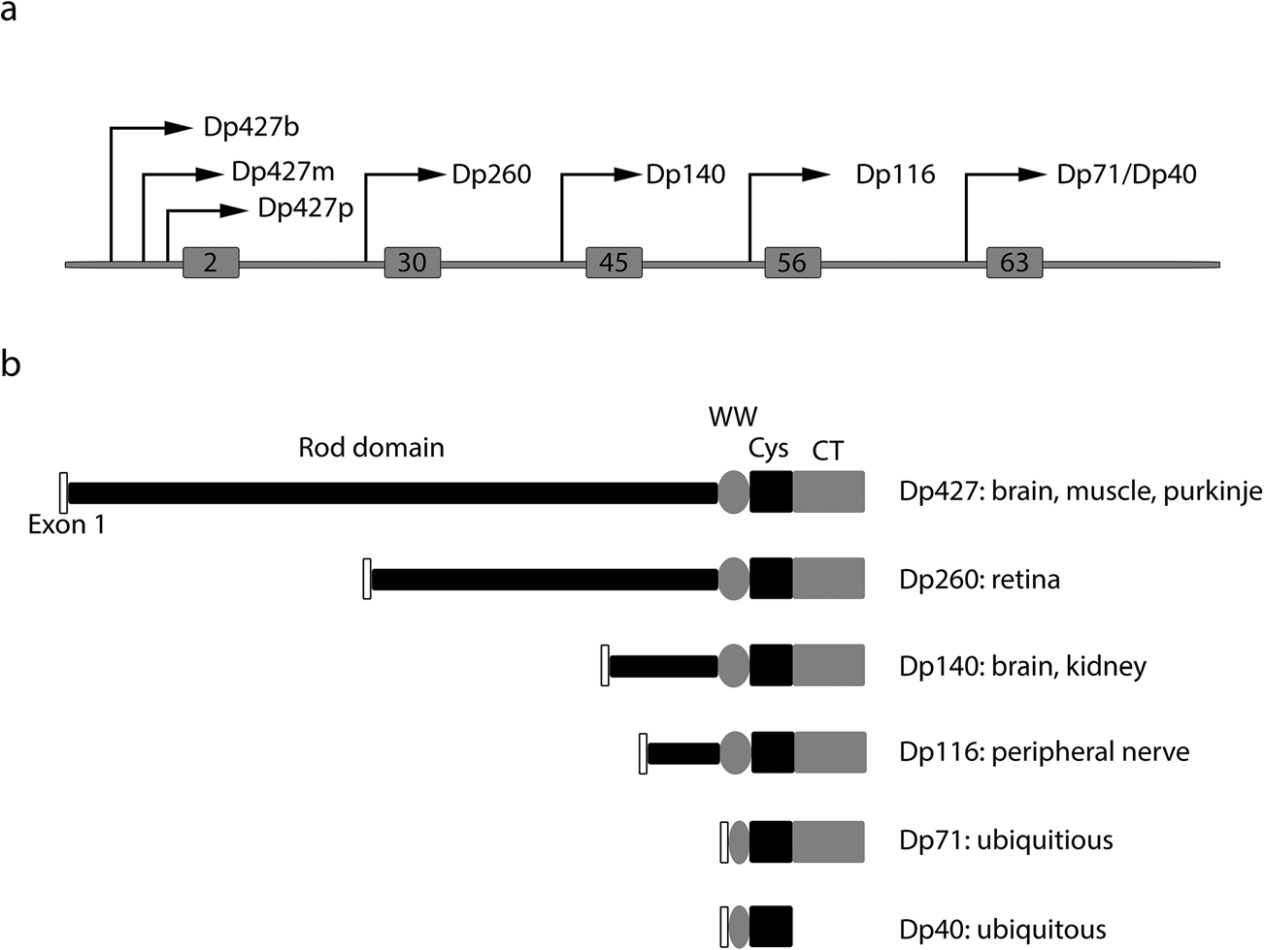
Structure of the *DMD* gene. **a** Schematic diagram showing the location of the seven independent promoters within the *DMD* gene. **b** Differential expression and domain structure of the different gene products, including unique first exons. Dp71 and Dp40 share a common promoter and first exon, Dp40 is produced through the use of an alternative polyadenylation site. Note that additional splice isoforms of Dp427p, Dp260, Dp140 and Dp71 are not shown. WW: WW domain; Cys: cysteine rich domain; CT: C-terminal domain

The *DMD* gene consists of 79 exons. It is one of the largest human genes and is located on the short arm of the X chromosome at position Xp21.2-p21.1. Seven independent tissue-specific promoters, as well as an alternative polyadenylation site, drive the transcription of multiple dystrophin protein variants (Fig. 1) [1]. Full-length dystrophin contains several functional domains: an N-terminal actin-binding domain, a central rod domain, a WW domain, a cysteine-rich domain and a C-terminal domain (Fig. 1). The cysteine-rich domain contains EF hand and ZZ domains that contribute to the stability of the WW domain and the interaction of dystrophin with dystroglycan, a member of the DAPC. The shorter dystrophin protein variants do not contain the N-terminal actin-binding domain, but instead have a shorter rod domain. Dp71 and Dp40 additionally lack the rod domain and part of the WW domain (Fig. 1). Many *DMD* gene products are alternatively spliced to produce multiple splice isoforms. Outside skeletal muscle, the most predominant and ubiquitous *DMD* gene product is Dp71, of which at least 14 splice isoforms have been identified [2]. These have been organised into groups, with the most common being Dp71d group isoforms that contain exon 78 and Dp71f group isoforms that do not contain exon 78. The grouping denotes alternative C-termini [2]. Dp71 exhibits a large functional diversity and its loss in the brain of Duchenne patients is thought to be responsible for cognitive and behavioural comorbidities [2].

*DMD* is located within the low-expressing FRAXC common fragile site (CFS, large regions of profound genomic instability) [3]. Genes within CFSs are frequently deleted and/or altered in cancer. Most of these sites span extremely large genes, some of which are known tumour suppressors. Their inactivation in different cancers is deemed non-random [4]. A common basis may underlie the frequent occurrence of CFS/*DMD* rearrangements in germ cells of DMD patients and similar alterations found in cancer cells [5].

Although case reports of cancer in Duchenne patients are rare and confined to round cell tumours to date [6–14] (Table 1), a growing and intriguing collection of evidence implicates the *DMD* gene and/or its protein products in tumourigenesis. Between 2007 and 2011 several studies have emerged showing that muscular dystrophy mouse models are prone to develop spontaneous soft tissue sarcomas (STS) [15–17]. Since then, numerous reports, of increasing frequency, have characterised *DMD* gene mutations and/or expression changes associated with the development, progression and/or survival of patients with a wide range of cancers including sarcomas, carcinomas, melanomas, lymphomas and leukaemia’s, as well as brain tumours [18–26]. Although a question of cause or effect remains, it is now clear that a role for *DMD* in cancer extends beyond just myogenic or musculoskeletal tumours and that the ratio of Dp427 to Dp71 may have particular importance. Here, we comprehensively review the overlooked, but accumulating evidence supporting a role for the *DMD* gene and its variants in neoplastic disease.

**Table 1:** Reports of concomitant DMD and cancer

Report	Tumour type	*DMD* mutation causative of Duchenne	Age at cancer diagnosis (years)	Outcome
Johnston et al. [12]	Stage III neuroblastoma	Unknown	0.75	Alive > 25 months post-surgery and chemotherapy treatment
Svarch et al. [13]	Acute lymphoblastic leukemia	Unknown	Unknown	Unknown
Rossbach et al. [8]	Stage IV ARMS	Deletion of exons 47–50	4	Alive after biopsy, chemotherapy and radiation treatment
Korones et al. [14]	Stage III Wilms tumour	Unknown	3	Alive six months after surgery, chemotherapy and radiation treatment
Jakab et al. [7]	Stage II ERMS	Unknown	7	Dead of disease 30 months after diagnosis
Saldanha et al. [9]	RMS	Unknown	5	Unknown post discharge after surgery
Doddihal and Jalali [6]	High risk medulloblastoma	Exon 44 deletion	7	Residual disease after surgery and radiation treatment, alive at 8 months post diagnosis
Büget et al. [10]	Massive RMS	Unknown	17	Unknown after discharge following left arm amputation
van den Akker et al. [11]	High risk medulloblastoma	Point mutation in exon 32 (c.4483C > T)	9	Alive 30 months post surgery, chemotherapy and radiation treatment with no residual or recurrent disease

## Sarcomas

Given the canonical role of dystrophin in muscle, it is unsurprising that a large body of evidence for a role for *DMD* in cancer comes from soft tissue sarcomas (STS), and the myogenic tumours in particular. We first review current knowledge on the incidence of STS in DMD patients and animal models before discussing wider evidence implicating *DMD* gene alterations in sarcoma patients.

### Evidence from DMD patients and animal models

We are aware of four clinical case reports (Table 1) on the concomitant occurrence of Duchenne muscular dystrophy (DMD) and rhabdomyosarcoma (RMS, the most common STS of childhood) [7–10]. The literature has not yet extended beyond this to investigate the true incidence and risk of STS in DMD patients. Reports of spontaneous tumour formation in muscular dystrophy mouse models, however, have gained more traction [15–17, 27] and have been reviewed in [28].

Chamberlain et al. were the first to report that older *mdx* mice (above 16 months of age) are prone to develop RMS-like tumours of the alveolar type [15]. The *mdx* mouse is a widely used non-transgenic DMD mouse model with a naturally occurring mutation (nonsense point mutation [C-to-T transition] in exon 23) abolishing Dp427 expression. RMS are extremely rare to non-existent in non-transgenic mice beyond *mdx* and are thought to only occur after genetic manipulation of the mouse genome [15]. Chamberlain et al. postulated that the increased incidence of RMS among *mdx* mice is due to the characteristic lifelong degeneration and regeneration of myofibres. This results in over-activation and proliferation of satellite cells and an increased risk of spontaneous mutations affecting the differentiation of their progeny [15]. This is in contrast to DMD patients, where the incidence of RMS appears lower, seemingly as a result of a significant drop in muscle mass and satellite cell numbers and their premature proliferative senescence [29]. In support of this, some studies have identified satellite cells as a potential origin for RMS [30].

Other studies have since confirmed the propensity of animal models lacking components of the DAPC to develop tumours. For example, dystroglycan abnormalities have been linked to cancer [16, 31, 32]. Dystrophin complexes with dystroglycan and loss of dystrophin is known to affect the level and localisation of other DAPC components [33]. In line with the results of Chamberlain et al., Fernandez et al. reported spontaneous development of RMS in 9% of *mdx* mice aged to over one-year-old, whilst none of the 450 similarly aged wild-type mice from their study developed tumours [16]. Fernandez et al. further characterised the RMS tumours in *mdx* mice and found that they are consistent with embryonal RMS (ERMS) rather than alveolar RMS (ARMS) as per the Chamberlain study [16]. They also found that *mdx* ERMS tumours showed altered expression patterns and mutations of known equivalent to human RMS-associated genes such as *TP53* and mouse double minute 2 ortholog (*MDM2*), a negative regulator of p53 [16].

A tumour suppressor role for dystrophin in mice is supported by another study by Schmidt et al. reporting a much higher incidence of mixed soft tissue tumours, including ERMS, in almost 40% of *mdx* mice [17]. It is unknown if this higher incidence is due to environmental factors. This study highlights the complex heterogeneous nature of STS tumours in *mdx* mice, with Chamberlain et al. and Fernandez et al. also reporting different histological subtypes. Schmidt et al. studied the incidence and onset of tumours in *mdx* (C57BL/10 background), *mdx3cv* (C57BL/6 background and deficient in C-terminal dystrophin gene products) and novel inbred strains revealing a strain-independent tumour suppressive role for dystrophin. However, the age of onset and incidence was strongly strain-dependent, with the C57BL/10 background of *mdx* mice being the most tumour-susceptible [17]. The additional loss of other muscular dystrophy-associated genes such as dysferlin and calpain-3 in double-mutant mouse lines was found to reduce sarcoma latency [17]. Another independent study reported RMS in > 90% of dystrophin and dysferlin double mutant mice [27]. Genomic analysis of solid tumours from *mdx* mice revealed genomic instability, DNA damage and frequent amplification of the *Met* or *Jun* oncogenes, loss of *Cdkn2a* and *Nf1* tumour suppressor genes and recurrent duplications of chromosomes 8 and 15, with chromosome 15 duplication being the more preferable duplication [17]. Interestingly, such genetic lesions are present before sarcoma development in aged *mdx* mice and, importantly, DMD patient skeletal muscle tissues also display gross genomic instability [17]. Adding to earlier theories, Schmidt et al. suggested that the adipocyte and connective tissue proliferation characteristics of DMD may create the required molecular context for sarcoma development from multipotent mesenchymal or muscle-derived stem cells.

A more recent study showed that, in a severe DMD mouse model, RMS formation and latency was affected by disease severity and that muscle stem cells from these mice can give rise to tumour spheres *in vitro* and RMS tumours *in vivo* [34]. Taken together, these studies indicate that the degenerative muscle environment of Duchenne patients (which has been associated with increased genetic instability in whole muscle) may promote the development of RMS through increased tissue turnover. This work highlights caution to be taken for the development of DMD therapeutics that act on the self-renewal ability of muscle stem cells.

### Evidence from human STS tissues

Outside the context of Duchenne muscular dystrophy, evidence for the involvement of the *DMD* gene in STS development is growing. A seminal study by Wang et al. cemented Dp427 as a tumour suppressor in myogenic tumours, where intragenic deletion of the *DMD* gene is a frequent mechanism by which they progress to high-grade lethal sarcomas [18]. This added to existing knowledge that the *UTRN* gene, which codes for the dystrophin-related protein utrophin, also acts as a tumour suppressor [35]. Using genome-wide single nucleotide polymorphism (SNP) assays, Wang et al. found intragenic *DMD* deletions in 63% of high-grade myogenic cancers, including gastrointestinal stromal tumour (GIST), leiomyosarcoma (LMS) and RMS. These deletions were not found in surrounding non-neoplastic tissues suggesting a somatic origin, and their frequency was higher in myogenic tumours compared to non-myogenic sarcomas or non-sarcoma cancer cell lines [18]. Further exploration using multiplex ligation-dependent probe amplification (MLPA)-based copy number assessment for each exon revealed *DMD* deletions in 43% of high-grade myogenic cancers [18]. These deletions are typically large, and although they vary between individuals, they usually begin with exons 1–3 and never extend past exon 62. As a result, all the deletions abolish Dp427 expression, which was absent or weakly present in 96% of metastatic GIST, 62% of metastatic LMS and 100% of metastatic RMS. Dp427 was strongly expressed in normal and benign counterparts for all tumour types studied. Interestingly, Wang et al. reported that expression of the smaller *DMD* gene product, Dp71, is maintained in cancers with *DMD* deletions. Knockdown of Dp71 in *DMD*-deleted RMS cells inhibited cell growth, suggesting that Dp71 expression is a requirement for myogenic tumours [18]. Re-expressing Dp427 using a mini*DMD* construct lacking exons 17–48 inhibited migration, invasion and invadopodia formation in multiple myogenic sarcoma subtypes, lending support to the hypothesis that depletion of Dp427 promotes the metastatic potential of myogenic tumours [18]. Indeed, Wang et al. reported that the same *DMD* deletions from one metastasis can be seen in all other metastases from the same patient. The authors suggested that *DMD* inactivation may be a driving event in myogenic cancer development and a target for therapeutic attack.

Subsequent work on GIST (the most common sarcoma of the gastrointestinal tract) confirmed an association between the dysregulation of dystrophin and GIST progression. Firstly, in a commentary in 2015, Wang and Fletcher [36] suggested, based on their work that *DMD* inactivation is a late-stage event enabling metastatic spread in GIST. GISTs largely result from constitutively activating mutations in KIT or PDGFRA receptor tyrosine kinases. Mutations disrupting the regulation of the cell cycle may serve as subsequent drivers of tumour progression, resulting in low-risk and high-risk GIST classifications. *DMD* inactivation adds a final step to a model for metastatic spread of myogenic cancers [36]. Additionally, another group confirmed the presence of *DMD* deletions in an Italian GIST series. In this study *DMD* deletions were only found in *KIT/PDGFRA* mutant GISTs (31% vs. 0% in wild-type), supporting the idea of *DMD* inactivation as a late event in metastatic progression [37]. Interestingly, the deletions identified in this study also involved the 5’ region with exons 2–7 being recurrently affected.

More recently, Mauduit et al. analysed 318 sarcomas using array CGH and confirmed the occurrence of *DMD* deletions in 16.5% of all types examined, 16.5% in sarcomas with complex genomic profiles (including LMS), 21.6% in synovial sarcomas and 14.2% in GISTs. The deletions were associated with metastatic development and decreases in *DMD* gene expression. In line with previous work, these deletions appeared to be restricted to the 5’ region of *DMD* and to affect the expression of the full-length Dp427 gene product. RNA sequencing analysis revealed that Dp71 was the only *DMD* gene product left intact [38]. Mauduit et al. for the first time found, using metastasis-free survival analysis, that intragenic *DMD* deletions are associated with a poorer prognosis, indicating that *DMD* deletion is an independent biomarker of metastatic evolution. Conversely, the authors found that neither Dp427 nor Dp71 expression levels were prognostic for metastatic progression, suggesting a central role for Dp427 deletion in this process [38]. Subsequent *in vitro* studies revealed, however, that Dp427 downregulation using CRISPR/Cas9 had no effect on the proliferation, migration or clonogenic properties of LMS cells [38]. In contrast, Dp71 downregulation using shRNA in synovial sarcoma and LMS cells that express Dp71, but not Dp427, did show a diminished proliferation, an abolished ability to grow as isolated clones and G2/M cell cycle phase arrest [38]. Dp71d (containing exon 78) plays a known role in cell division, where it is localised at the mitotic spindle poles of rat pheochromocytoma PC12 cells and acts through interaction with lamin B1 and β-dystroglycan [2, 39]. Inhibition of Dp71 likely induces cell cycle arrest, which may explain the requirement of Dp71 for tumour progression. Dp71 is, therefore, an attractive therapeutic target for Dp427-depleted sarcomas.

Inactivation of tumour suppressor genes through homozygous deletion is common in cancer. Intron retention is another common mechanism by which tumour suppressor genes may be inactivated [40]. As such, *DMD* intron retention cannot be ruled out as a potential mechanism for Dp427 inactivation in some cancers. Evidence for this notion came from Niba et al. who assessed dystrophin expression in the ARMS-derived cell line CRL-2061. These cells do not contain full-length dystrophin protein despite the presence of a full and structurally normal *DMD* gene and full-length mRNA expression. In this cell line, three retained introns (40, 58 and 70) accumulating in 90% of *DMD* transcripts are present, which are thought to abolish dystrophin expression through the introduction of stop codons and, thereby, disruption of the tumour suppressive role of Dp427 [41]. Dp71 was not studied by Niba et al. who nonetheless suggested that post-transcriptional *DMD* modifications should be considered as secondary alterations in cancer. Cancer-specific vulnerabilities can be exposed when a deleted tumour suppressor gene is part of a family of functionally redundant genes [38, 42]. In the case of *DMD* inactivation in sarcomas, metastatic progression may be determined by the ratio of Dp427 to Dp71, a suggestion that warrants further investigation.

## Tumours of the nervous system

The studies by Wang et al. described above indicate that *DMD* deletions are infrequent in non-myogenic tumours, but they did not fully analyse *DMD* mRNA expression levels across other tumour types. Luce et al. investigated *DMD* expression levels and genetic alterations in non-myogenic tumours in pairwise comparisons to normal tissues. They studied 16 tumour types using microarray-based gene expression and RNAseq datasets from the GEO database and cBioPortal public repositories, respectively. The RNAseq data showed that the median frequency of *DMD* alterations in non-myogenic tumours (3.4%) was higher than that in other common tumour suppressor genes, such as *BRCA1* (1.6%), *BRCA2* (2.8%) and *PTEN* (3%). Several cancers of the nervous system were included in this study, revealing that *DMD* is significantly overexpressed in ependymoma and astrocytoma, but significantly under-expressed in medulloblastoma and non-significantly under-expressed in glioblastoma [43]. The authors speculated that these findings may be attributed to Dp71, as it is the predominant *DMD* product in the brain and has been shown to play a role in proliferation, invasion and migration [2, 24, 44]. However, the study did not differentiate the expression of different isoforms and no immunohistochemistry was performed to correlate the findings to protein expression.

### Neuroblastomas

Other studies have investigated the role of *DMD* gene deletions in neuroblastomas using patient samples. Gallia et al. performed whole exome sequencing (WES), whole genome sequencing (WGS) and SNP array analysis on 14 olfactory neuroblastoma patient samples and found that somatic *DMD* deletions occurred in 86% of the tumours [22]. In line with *DMD* deletions in STS, in olfactory neuroblastoma these are concentrated in the 5’ end of the gene and all but one are predicted to preserve Dp71 expression. Numerous *DMD* transcripts were encountered in the neuroblastoma cell line SH-SY5Y and, as observed in an ARMS cell line described above [42], *DMD* transcripts in SH-SY5Y cells showed retention of part of intron 40, implying that this event may be cancer-specific and represent another means of *DMD* dysregulation in neurological cancers [45].

### Meningiomas

The findings in olfactory neuroblastoma are supported by a similar study evaluating *DMD* gene deletions in high grade/progressive meningioma patient samples [46]. Of the 55 patients with progressive/high grade tumours evaluated, 17 (32%) either exhibited deletions in the *DMD* gene or silenced expression. Somatic *DMD* deletions were found in 5/24 patient samples (20.8%), spanning exons 2–30 in three male patients and the entire gene in two female patients. Further genome-wide analysis of fragile sites and 40 other large genes indicated that these do not represent general non-specific genome instability [46]. Western blotting and immunohistochemistry on patient tissues harbouring *DMD* deletions confirmed a loss or reduction of Dp427 protein expression, and electron microscopy revealed a reduced density of cytoskeleton filaments in tumour cells. Interestingly, compared to *DMD* wild-type, patients with *DMD* alterations showed significantly shorter progression-free (1.6 years vs. 2.6 years, *p* = 0.038) and overall survival (5.1 years vs. median not reached, *p* = 0.006) times [46]. *DMD* alterations were also found to be significantly more common in progressive/high grade meningiomas than in grade I and II non-progressive meningiomas. This suggests that *DMD* inactivation may be associated with high grade malignancy in meningioma. Adding to evidence for a role for *DMD* in meningioma, Paramasivam et al. analysed mutation patterns and regulatory networks in meningioma tumour samples and found that *DMD* was the second most frequently altered gene (19% of cases) [47]. Of 12 samples harbouring *DMD* mutations, 11 had alterations in both copies of the neurofibromin 2 *(NF2)* gene, the most commonly mutated gene in meningioma, which encodes the tumour suppressor protein merlin. Expression analysis of methylation subgroups showed that *DMD* expression was higher in subgroups with *NF2* mutations compared to *NF2* wild-type cases [47]. *DMD* was also found to be one of the most highly upregulated genes in *NF2* mutant samples. Future functional work would be useful to identify specific roles of *DMD* variants in meningioma development.

### Glioblastomas 

As part of a study on the selective loss of large CFS genes in cancer, McAvoy et al. showed that *DMD* expression (by qRT-PCR using primers in the 3’-untranslated region) is reduced in all brain tumour cell lines tested, as well as in a xenograft derived from an intracranial model of glioblastoma (GBM) [3]. A complex picture of DMD, and likely DAPC, disruption within nervous system tumours is apparent since alterations in the dystrophin-associated protein α-dystroglycan have also been reported in human gliomas [48]. The major dystrophin protein in the brain is Dp71 and it exhibits a large functional diversity. Evidence supports the presence of functional Dp71-containing DAPCs in the brain, but the individual components may vary according to cell type [2]. Of note, Dp71 plays a role in maintaining blood brain barrier integrity, which is disrupted in human gliomas as well as in the *mdx* mouse model [2, 49]. To date, six Dp71 splice isoforms have been identified in the U251-MG glioblastoma cell line and, as yet, their specific functions are unclear [50]. There is value to investigating the effects of individual dystrophin proteins on neurological cancers, in addition to overall *DMD* expression and mutation analyses. Ruggieri et al. looked specifically at Dp71 in human GBM, the most aggressive malignant CNS tumour, and meningioma. They found that expression attributed to Dp71d was increased in a meningioma cell line and decreased in GBM cell lines and in human GBM biopsy specimen cells compared with a normal human astrocyte cell line [23]. They also showed that a higher Dp71 expression was associated with a lower Ki-67 proliferative index in GBM patients, which may suggest that increased Dp71 expression is associated with reduced proliferation.

### Summarising DMD dysregulation in nervous system tumours

Taken together, these results highlight some inconsistencies when evaluating total *DMD* expression in cancer, as high *DMD* expression may be associated with the progression of certain cancers whereas low expression may be associated with the progression of others. As with STS, the ratio of Dp427 to Dp71 may be a pertinent factor and, thus, differentiating between these isoforms in mRNA and protein expression studies is important. Furthermore, it is possible that different tumours and/or tissues express a different complement of Dp71 isoforms which may serve to either supress or progress tumour development. A fuller understanding of dystrophin biology in the brain is required. It has been reported that a complex DMD neuropsychiatric syndrome arises from the absence of distal *DMD* gene products in the brain of Duchenne patients [2]. This indicates that altered *DMD* gene products in the brain can disrupt nervous system function regardless of cancer. Of note, there is a case report of neuroblastoma in DMD [12], as well as two other case reports describing medulloblastoma in children with DMD (Table 1). The former described a seven year old boy with a *DMD* deletion of exon 44 and the latter a nine year old boy with a *DMD* point mutation in exon 32 with anaplastic medulloblastoma [6, 11]. The central location of both of these mutations in the dystrophin gene suggests that Dp71 is unaffected and that the individuals are not expected to display severe intellectual disability based on our knowledge of the genotype to phenotype ratio in DMD [2]. The ratio of Dp427 to Dp71 would presumably, however, be disrupted.

## Melanomas

Downregulation of *DMD* has first been implicated in the pathogenesis of malignant melanoma by Kӧrner et al. [21]. Digital karyotyping and multiplex PCR analysis of cell lines derived from established metastatic melanomas revealed *DMD* deletions in the M1 (in-frame deletion of exons 3–29), RPMI-7951 (in-frame deletion of exons 17–30) and WM-793 (out-of-frame deletion of exons 42–43) cell lines [21]. These deletions are outside the major hotspot region of exons 45–53 for *DMD* mutations in Duchenne muscular dystrophy patients [51, 52] and appear to be tumour-specific mutational events [21]. *DMD* expression analysis of full-length Dp427 variants in non-cancerous primary melanocytes from three different donors revealed high expression levels of Dp427m [21]. Conversely, in a panel of 55 melanoma cell lines, Dp427m expression was found to be very low in 18% of the lines and absent in 69% of them. In comparison to normal melanocytes, 32/37 melanoma cell lines exhibited a reduced *DMD* expression [21]. Additionally, the same melanocytes also expressed Dp71 and Dp116. Western blot analyses revealed that Dp71 expression was maintained independent of that of Dp427 in 100% of the melanoma cell lines analysed, whereas Dp116 expression was only present in 20% of cell lines negative for Dp427m. This is akin to STS and nervous system tumours, where the balance of Dp427 and Dp71 has also been found to be disrupted.

To further investigate the decreased *DMD* expression observed in malignant melanoma, *DMD* RNA was isolated and cDNA sequenced from 34 melanoma cell lines (25 negative and nine positive for full-length dystrophin) and three primary melanomas with low *DMD* mRNA expression levels [21]. The sequencing results revealed five known polymorphisms and six new variants including D214N (MM-232 cell line) located within the actin-binding domain and G3189E (WM1205LU cell line) located within the C-terminal ZZ domain [21]. These variants are considered to be melanoma-specific since no new variants were found in cell lines expressing Dp427m that may affect dystrophin function. Indeed, there is evidence for a functional DAPC in melanocytes [21] and the importance of dystrophin for melanocyte cell function warrants further investigation.

Knockdown of Dp427m in melanoma cell lines (M7 and C-32) resulted in reduced spheroid formation and enhanced migration and invasion capacities [21]. Kӧrner et al. also restored dystrophin expression in cell lines with no detectable dystrophin protein using a GFP-tagged full-length dystrophin expression construct. Decreased migration, reduced proliferation and induction of cellular senescence were observed in the restored cell lines compared to the respective controls [21]. The latter effect on cellular senescence is a common phenomenon after tumour suppressor gene expression restoration, further supporting a role for *DMD* in tumour suppression [53, 54]. The findings of Kӧrner et al. are supported by a bioinformatics study of Luce et al. who reported that *DMD* expression is lower in melanoma tissues in comparison to normal skin tissues [43].

## Haematological malignancies

### Lymphomas

Lymphomas and lymphocytic leukaemia’s have both been linked to aberrant *DMD* gene expression [43]. Baumforth et al. showed, using microarray-based analyses (with a *DMD* probe set that detects all *DMD* transcripts) that *DMD* is downregulated 8-fold in primary Hodgkin’s lymphoma (HL, nodular sclerosing subtype) tumour tissues compared to germinal centre B cells [19]. This finding was supported by Luce et al. who similarly observed downregulation of *DMD* in lymphomas vs. normal centroblasts and centrocytes [43]. The oncogenic herpesvirus, Epstein-Barr virus (EBV), is present in approximately half of HL tissues and cells. Baumforth et al. found that *DMD* expression is upregulated in EBV-positive HL tissues and cells compared to non EBV-positive HL tissues and cells (1.66-fold in tissue and 1.64-fold in L591 cells) [19]. These findings warrant further investigation to explore the potential oncogenic role of EBV-induced *DMD* upregulation in lymphomas and whether EBV is associated with differential expression of dystrophin in other EBV-associated malignancies. Interestingly, there have been two reported case studies of young boys with Hodgkin’s disease and non-Hodgkin’s lymphoma (a large B-cell lymphoma of the ascending colon) associated with Becker muscular dystrophy, the milder allelic form of DMD caused by in-frame *DMD* deletions [55, 56].

### Leukaemia’s

One case report of DMD concomitant with acute lymphoblastic leukaemia has so far been published [13] (Table 1) and several independent studies using microarray-based gene expression profiling have highlighted the diagnostic potential for *DMD* in B-cell chronic lymphocytic leukaemia (B-CLL) [57–59]. Nikitin et al. further reported that high *DMD* expression across 134 B-CLL patients was associated with a shorter lymphocyte doubling time and was predictive of a poor patient survival (median overall survival for patients with high *DMD* expression was 90.1 months vs. median not reached for patients with low *DMD* expression) [20]. These observations are in contrast with the trend towards a tumour suppressive role for *DMD* in other tumours, although this does appear to be restricted to Dp427. We note that the primer and probe set used in this study spans exons 66–68 and will, therefore, detect both Dp71 and Dp427 expression. The increased *DMD* expression in B-CLL can, therefore, not be attributed to either Dp427 or Dp71. The increased expression has, however, been found to be associated with the mutation status of immunoglobulin variable region genes (IgVH) (*p* < 0.0001), which is considered the strongest long-term predictor of prognosis in B-CLL [20]. As with other tumour types, it remains to be seen whether *DMD* plays a key role in the pathogenesis of B-CLL or whether its altered expression represents a secondary event. Interestingly, reduced dystroglycan expression has previously also been linked to the pathophysiology of leukaemia [60].

## Carcinomas

### Lung adenocarcinomas

Dp71 has been reported by Tan et al. to have an oncogenic role in lung adenocarcinoma cells using several *in vitro* functional assays. The authors silenced total Dp71 (albeit, non-specifically since the target was exon 69 present also in Dp427) using shRNA in A549 cells and found that the Dp71-depleted cells grew, migrated and invaded significantly slower than the empty vector control cells [24]. Cisplatin-induced apoptosis was also found to be increased in Dp71-depleted cells via enhanced caspase activity. Furthermore, *in vivo*, a xenograft mouse model established with Dp71-depleted cells showed reduced tumour growth compared to controls. The tumours expressed lower levels of lamin B1 (a Dp71-binding partner), matrix metalloproteinase 2 (MMP-2) and B cell lymphoma 2 (Bcl-2) [24], which the authors linked to the reduced malignancy in this model.

Subsequently, Luce et al. reported significantly reduced *DMD* expression in lung adenocarcinoma and non-small cell lung carcinoma tissues vs. normal lung tissues using data obtained from the GEO repository with a probe set that detects all *DMD* transcripts [43]. Given the findings of Tan et al. and the evidence from other tumours discussed in this review, it is possible that the reduced expression observed by Luce et al. relates to reduced Dp427 expression, but further study is needed to confirm this hypothesis.

### Gastric adenocarcinomas

In contrast to the oncogenic role of Dp71 in lung adenocarcinomas as discussed above, a second paper by Tan et al. reported tumour suppressive associations of Dp71 expression in gastric adenocarcinomas. Reduced Dp71 protein and mRNA expression levels were found in ~ 70% of primary gastric adenocarcinoma patient tumour tissue samples compared to matched non-tumour tissues [25]. We note, however, that the antibody and primers used in this study cannot distinguish between Dp427 and Dp71, except on Western blots where a reduction in Dp71 in tumour tissues was evident. Immunohistochemical analyses also revealed that cancer cell differentiation and lymph vascular invasion were significantly associated with Dp71 downregulation. In line with this finding, Kaplan-Meier analysis of patients after surgical resection revealed that those with a high Dp71 expression exhibited a significantly favourable overall survival time compared to those with a low Dp71 expression (56.56% vs. 30.8% 5-year survival rate, respectively; *p* < 0.001) providing evidence that Dp71 may act as a tumour suppressor in gastric adenocarcinoma [25]. To support this notion, Tan et al. overexpressed either Dp71d or Dp71f in gastric cancer cells (which also exhibited diminished Dp71 expression) and observed inhibition of proliferation in the transfected cells compared to controls. Additionally, co-immunoprecipitation analysis confirmed an interaction between Dp71 and lamin B1 in normal human gastric epithelial cells, supporting previous findings and suggesting a critical role for Dp71 in cell division and a potential importance of the Dp71-lamin B complex in tumour suppression [25, 39].

### Carcinomas of the head and neck

Nasopharyngeal carcinoma (NPC) originates from epithelial cells within the nasopharynx. Its incidence is highest among males and its occurrence is particularly frequent in China. An X chromosome-wide association study for SNPs in 1590 Chinese NPC patients and 994 controls revealed an association within the *DMD* gene (intronic SNP rs5927056, *p* = 1.49 × 10^− 5^), which was validated in Taiwanese and Malaysian replication cohorts [26]. Combination analyses revealed a consistent association in males and showed that the *DMD* SNP rs5927056 is associated with a reduced risk of NPC (odds ratio: 0.85, *p* = 9.44 × 10^− 5^) [26]. Additionally, NPC risk association with X chromosome SNPs was found to be decreased by 8.3% after excluding rs5927056, suggesting a strong effect of this locus on NPC susceptibility.

Additional RNAseq and qRT-PCR analyses of oropharyngeal squamous cell carcinoma (OPSCC) tissues revealed *DMD* as one of a number of large CFS genes to be significantly downregulated in tumour tissues [61]. Decreased expression of a select group of six genes, including *DMD*, was found to be associated with an increased recurrence of OPSCC [62]. The prognostic utility of *DMD* alone was not studied.

### DMD across other carcinomas

Next to the carcinomas discussed above, Luce et al. reported downregulated *DMD* expression in primary and metastatic prostate cancers, pancreatic ductal adenocarcinomas and colon and breast cancers. In case of prostate cancer, the expression of *DMD* decreased further when the cells became metastatic. This observation was consistent with that in melanomas, which showed a decreased expression compared to that in benign nevi, which in turn showed a decreased expression when compared to normal skin tissue. Interestingly, in sporadic breast cancer, the median frequency of *DMD* alterations (3.95%) was higher than those of *BRCA1* (1.95%) and *BRCA2* (3.4%) [43]. Additional survival analyses revealed that in breast and uterine cancer, patients with *DMD* alterations exhibited significantly poorer survival rates compared to wild-type patients [43]. Overall, with the exception of renal cell carcinoma in which *DMD* is upregulated [43], there is a trend for *DMD* downregulation across numerous carcinomas (Table 2). The mechanism and impact on individual gene products remains, however unclear. For some carcinomas, the *DMD* gene and/or its protein products appear to be tumour suppressive, whereas in others they appear to be oncogenic. Dp71 in particular elicits opposing effects in gastric and lung adenocarcinomas. These observations highlight a need to determine the full complement of *DMD* transcripts expressed in tumours and corresponding normal tissues. Of note, expression of the dystrophin-associated protein β-dystroglycan has also been found to be reduced across several carcinomas, and its function has been linked to the development and progression of prostate cancer [63–65].

**Table 2:** Summary of evidence for a role of the *DMD* gene in cancer

Tumour type	*DMD* mutation(s)	*DMD* expression	Oncogenic or tumour suppressive?	Strength of evidence	Ref(s)
STS	5’ deletions affecting only Dp427. Intron retention.	Dp427 absent or severely reduced, Dp71 maintained	Tumour suppressive role for Dp427	+++	[18, 37, 38, 41]
Olfactory neuroblastoma	5’ deletions	Predicted to affect Dp427 expression and maintain Dp71	nd	++	[22]
Meningioma	5’ deletions, 2nd most frequently altered gene	Reduced Dp427 expression	Tumour suppressor role for Dp427	+++	[46, 47]
Malignant melanoma	Tumour specific deletions and polymorphisms e.g. IF Δ3–29, IF Δ17–30, OOF Δ42–43	Reduced Dp427m expression, Dp71 maintained, Dp116 frequently absent	Tumour suppressor role for Dp427	+	[21, 43]
Lymphoma	nd	*DMD* downregulated in tumour vs. progenitor cells. *DMD* upregulated in EBV-positive vs. EBV-negative tumours	nd	++	[19, 43]
Lymphocytic leukaemia	nd	*DMD* upregulated	Oncogenic	++(+)	[20, 43, 57–59]
Lung adenocarcinoma	nd	*DMD* downregulated	Oncogenic role for Dp71	++	[24, 43]
Gastric adenocarcinoma	nd	*DMD* downregulated, attributed to Dp71	Tumour suppressive role for Dp71	++	[25]
Nasopharyngeal carcinoma	Intronic SNP rs5927056	nd	nd, SNP associated with reduced risk	++	[26]
Oropharyngeal squamous cell carcinoma	nd	*DMD* downregulated	Tumour suppressive	++(+)	[61, 62]
Renal cell carcinoma	nd	*DMD* upregulated	nd	+	[43]
Other carcincomas including prostate cancer, pancreatic ductal adenocarcinoma, colon, breast and uterine cancer	nd	*DMD* downregulated (nd for uterine cancer)	Tumour suppressive	+(+)	[43]

Only characterised mutations are noted. Where we refer to *DMD* we cannot attribute the effect to specific gene product(s). IF: in-frame; OOF: out-of-frame; Δ: denotes exon deletions; nd: not determined; +: *in-vitro* or *in-silico* evidence only; ++: primary tumour cells or *in-vivo* evidence, tissue gene expression; +++: survival and clinicopathological associations, xenograph models. Parentheses indicate evidence partially met

## Conclusions and perspectives

**Fig. 2:**
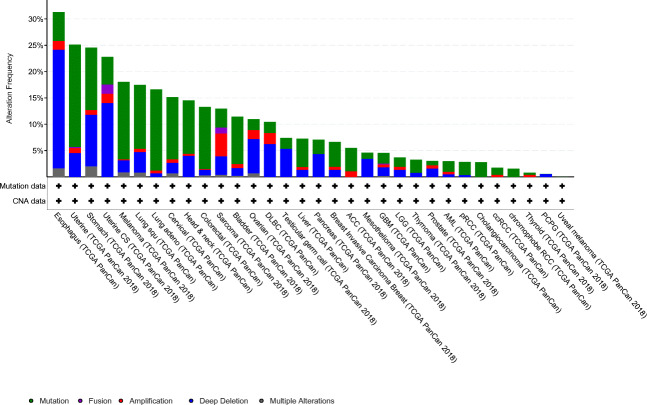
cBioportal was used to rank *DMD* alteration frequencies across the TCGA PanCancer Atlas studies (10,953 patients across 32 studies). Alteration frequencies consist of mutations (green), fusions (purple), amplifications (red), deep deletions (blue) and multiple alterations (grey). Only TCGA studies with both mutation data and copy number alteration (CNA) data are shown

**Fig. 3:**
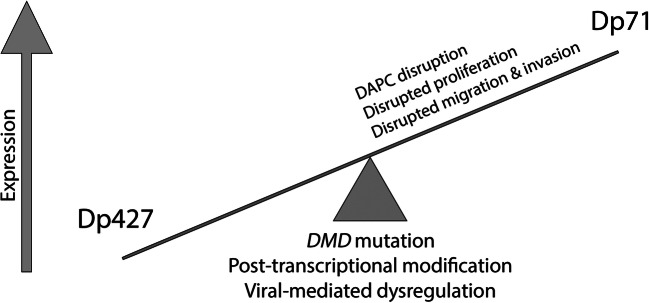
Proposed model of *DMD-*driven cancer development. The relative balance of the Dp427 and Dp71 gene products influences the progression to full neoplastic disease. Altered *DMD* gene product levels have tissue-specific effects on cancer hallmarks, such as proliferation and invasion, and disrupt the dystrophin-associated protein complex (DAPC)

The known contribution of *DMD* dysregulation in the pathogenesis across several cancers has been highlighted. There is striking evidence for a role for *DMD* in most major tumour types: sarcomas, leukaemia’s/lymphomas, melanomas, carcinomas and nervous system cancers. However, some key questions remain. Firstly, the incidence and risk of cancer for DMD patients is unknown, in particular whether germline *DMD* inactivation may predispose to sarcoma. This will become increasingly important as individuals with DMD are living longer due to improved standards of care, and novel treatments under development will not necessarily mitigate against cancer risk. For example, life-lengthening treatments that do not slow ongoing myofiber necrosis and regeneration might increase the risk of RMS in older patients [15]. An increased risk of cancer in myotonic dystrophy has been revealed through a population-based survey [66], but to our knowledge this has not been done for DMD patients. Knowledge of risk may allow for individuals to receive risk counselling. Secondly, there is little evidence across different cancers that *DMD* expression and/or mutation status may serve as independent prognostic biomarkers. Future studies should address this question and whether *DMD* acts as a driver or passenger in tumourigenesis; the question of cause or effect remains. The frequency of *DMD* gene alterations (i.e., mutations and copy number alterations) varies across cancers (Fig. 2 and [43]). In some cases, there is clear evidence of recurrent mutations that abrogate the expression of *DMD* gene products (i.e., Dp427 in STS) and/or specific functional domains such as the N-terminal actin-binding domain as seen in meningioma [46]. Disrupted or altered gene expression through non-mutational mechanisms may also be relevant for disease development (i.e., virus-induced alterations in gene expression programmes or epigenetic modifications). A clear requirement for future work will be to identify which *DMD* gene products, and isoforms thereof, are present in individual tumours. The function of Dp71d and Dp71f group isoforms in cancer warrants particular investigation given conflicting data on a role in cancer cell proliferation [23, 38]. Single cell or bulk RNA next generation sequencing may be informative in this regard, as well as the development of antibodies specific for the individual gene products. We suggest that the ratio of Dp427 to Dp71 may be important in disease progression rather than their absolute levels (Fig. 3). The tumour context may be important with gene variants acting as either oncogenes or tumour suppressors depending on the specific tumour microenvironment. To our knowledge a role for *DMD* in intra-tumour heterogeneity has not been examined, and more work is required to ascertain the mechanisms by which *DMD* dysregulation alters cancer cell functions.

Also, several cellular pathways implicated in cancer development are disrupted in DMD. These include the Hippo signalling pathway, which regulates apoptosis, proliferation and tissue homeostasis. The expression of the downstream target of the Hippo pathway, yes-associated protein 1 (YAP), is decreased in DMD muscle [67]. In the context of Duchenne, activation of YAP is beneficial for stimulating tissue repair, whilst its inhibition is being pursued as an anticancer strategy [67]. Another example comes from studies on telomeres, the maintenance of which is important in cancer. An age-dependent telomere shortening occurs in DMD muscle, which has been linked to cardiac failure [68–71]. This shortening is associated with increased expression of telomeric repeat binding factor-1 (TRF1) and poly (ADP-ribose) polymerase-1 (PARP1), which modulate telomere elongation [68].

The *mdx* mouse represents a useful *in vivo* model to dissect the contribution of *DMD* to cancer development, since Dp427 expression is abolished while that of Dp71 is maintained. However, despite numerous studies acknowledging the increased, and unique, incidence of sarcomas in *mdx* mice, detailed analysis to fully understand this phenomenon is yet to be performed. We also advocate the development of additional models such as human xenograft models to assess the contribution of *DMD* to cancer development and the response to therapeutic regimens.

There are exciting prospects for future combination-based approaches that incorporate *DMD*-targeted therapy in cancer. Such approaches may involve altering the ratio of different *DMD* gene products, for example by restoring Dp427 expression or by reducing/abolishing the expression of Dp71. Recent drug approvals for Duchenne utilise splice switching antisense oligonucleotides to restore dystrophin expression in patients [72–74]. Such innovative approaches might be repurposed for the treatment of *DMD*-associated malignancies, but only when the underpinning role of *DMD* in tumour development has been fully established.

## Data Availability

Not applicable.
